# Aortic neck banding with endograft preservation for refractory type IA endoleak: The role of intraoperative duplex ultrasound

**DOI:** 10.1016/j.jvscit.2026.102284

**Published:** 2026-05-04

**Authors:** Andrea Ascoli Marchetti, Fabio Massimo Oddi, Manuel Francesco Romano, Cataldo Caruso, Mauro Fresilli, Martina Battistini, Stefano Fazzini, Eugenio Martelli

**Affiliations:** Vascular Surgery Unit, Department of Biomedicine and Prevention, University of Rome Tor Vergata, Rome, Italy

**Keywords:** Aneurysm, Aorta, Banding, Endograft preservation, Endoleak type I, Introperative ecocolor doppler, Ultrasound examination

## Abstract

A case of late type IA endoleak is reported. The endoleak occurred approximately 1 year after successful treatment of a type IB endoleak with iliac branch device implantation in a patient who had undergone endovascular aneurysm repair 10 years earlier for an abdominal aortic aneurysm with severe neck angulation. The patient underwent secondary endovascular revision, which proved ineffective, with persistence of the endoleak and progressive aneurysm sac enlargement. A minimally invasive open surgical approach with endograft preservation, consisting of external proximal aortic neck banding, was therefore undertaken. Complete resolution of the endoleak was achieved and confirmed intraoperatively by duplex ultrasound. This case highlights the role of aortic neck banding as an effective salvage strategy for type IA endoleak refractory to endovascular treatment and the potential role of duplex ultrasound as a diagnostic gold standard in the intra- and postoperative periods.

Endovascular aneurysm repair (EVAR) has become a cornerstone in the management of abdominal aortic aneurysms, offering reduced perioperative mortality and faster recovery when compared with open surgical repair. However, long-term durability is limited by late complications, especially in the case of neck angulation, which remains the predominant cause of reintervention and conversion to open surgery. Among the different types of endoleak, proximal sealing failure (type IA) is associated with the most severe clinical consequences.

Type IA endoleak, resulting from inadequate proximal endograft sealing, represents a particularly high-risk complication due to persistent aneurysm sac pressurization and the increased risk of aneurysm enlargement and rupture. Management is often challenging, especially in patients with hostile proximal neck anatomy or progressive post–EVAR aortic remodeling. Reported incidence ranges from approximately 1.2% to 3.7% within 2 years of follow-up, although late presentations related to progressive neck dilatation have also been described.^1^^,^^2^

When secondary endovascular repair fails or is not feasible, open surgical conversion is traditionally required and is associated with significant morbidity. External proximal aortic neck banding or banding has emerged as a less invasive alternative, enabling restoration of proximal sealing while preserving the endograft.

In this report, a case of post–EVAR endoleak evolution is described, initially type IB and subsequently type IA, successfully treated with external abdominal aortic neck banding with endograft preservation, and the surgical technique and its clinical rationale is discussed.

## Case report

**Fig 1:**
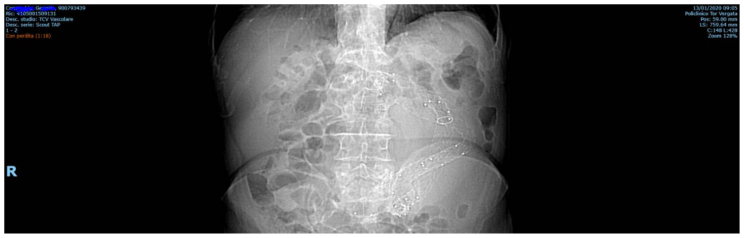
Direct X ray. Postoperative surveillance demonstrating the previous tortuosity. In the same period, computed tomography scan demonstrated no sign of endoleak.

An 89-year-old male former smoker with a history of coronary artery disease, arterial hypertension, and early-stage chronic kidney disease underwent EVAR in 2014 using an Endurant II endograft (Medtronic Inc) for a 6-cm abdominal aortic aneurysm. Computed tomography angiography (CTA) and X ray (Fig 1) showed no evidence of endoleak. Periodic imaging surveillance during follow-up demonstrated stable aneurysm sac dimensions without signs of endoleak.

**Fig 2:**
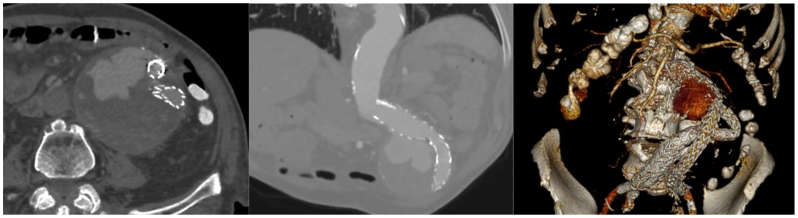
Computed tomography angiography (CTA) scan demonstrated a type Ib endoleak diagnosis: maximum intesity projection (MIP) and surface shaded display (SSD) show the contrast dine in the aneurismal sac.

In 2024, routine duplex ultrasound (DUS), followed by contrast-enhanced aortoiliac CTA, revealed a type IB endoleak. Secondary endovascular repair was performed with implantation of a right iliac branch device (GORE IBE; W.L. Gore & Associates) (Fig 2).

Approximately 9 months after iliac branch device implantation, during follow-up in 2025, CTA demonstrated rapid aneurysm sac enlargement from 6 cm to 10 cm, with contrast opacification consistent with a type Ib endoleak. Given the persistent sac pressurization and high risk of rupture, a secondary endovascular intervention was planned.

**Fig 3:**
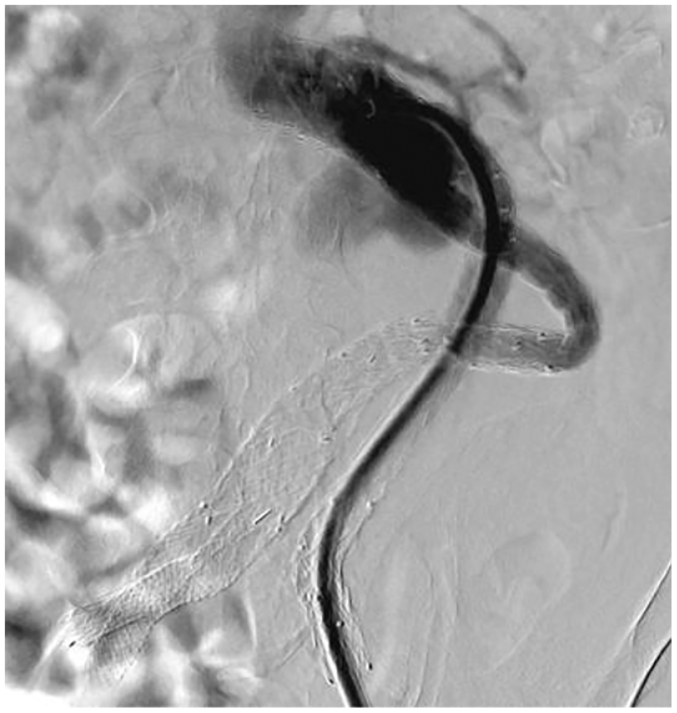
A postoperative type Ia endoleak is shown after treatment with endovascular procedures.

Secondary endovascular repair was performed using bilateral percutaneous femoral access and right humeral access. A proximal aortic cuff (Endurant, 36 × 45 mm; Medtronic Inc) was deployed, followed by left renal artery chimney stenting with a Dynetic Renal stent (6 × 19 mm). Completion angiography demonstrated patency of both renal arteries and the aortic endograft, with persistent proximal type IA endoleak. To improve proximal fixation, six Aptus Heli-FX EndoAnchors were deployed at the proximal covered segment of the endograft, resulting in further reduction of the endoleak (Fig 3). The procedure was completed without intraoperative complications.

At 15-day follow-up, DUS demonstrated persistence of the type IA endoleak with ongoing aneurysm sac pressurization. Given the failure of secondary endovascular repair, open surgical treatment was planned.

**Fig 4:**
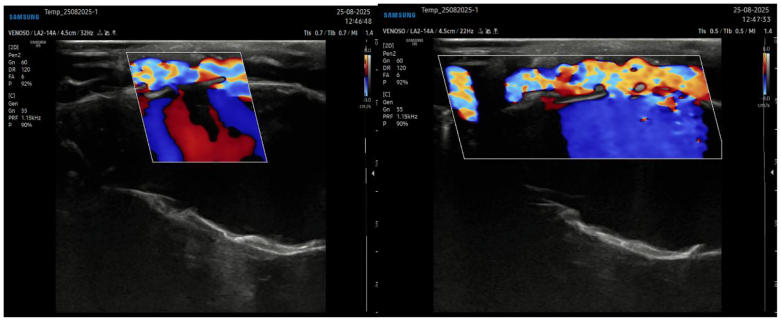
Intraoperative duplex ultrasound (DUS) examination. In the region of interest (ROI), the presence of flow in the aneurysmal sac is appreciate. The presence of flow in stent graft in the lower part is present.

**Fig 5:**
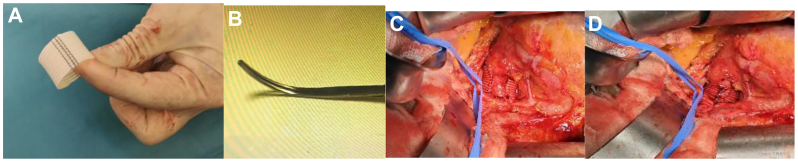
**A,** 28 mm Dacron woven, prosthesis used to make the bandage. **B,** Iliac clamp used for retroartic passage of the prosthesis. **C,** The prosthesis surrounds the neck of the aneurysm before suturing. **D,** The prosthesis was sutured around the neck.

The treatment of this condition was deemed appropriate only after a thorough discussion with the patient and relatives, given the low surgical impact without clamping or hemodynamic changes. A total surgical conversion would not have been indicated. The patient underwent a xipho-umbilical mini-laparotomy. The proximal aneurysm neck was exposed and appeared rightward convex with scoliotic configuration defined as a permanent lateral deviation of the aortic aneurysm course, which was not limited to a lateral displacement, but involved a three-dimensional structural deformity. The inflammatory reaction was less than expected and generally observed when preparing aortas for surgical conversion.^3^ In any case, we attempted to limit dissection to the bare minimum. The left renal vein was identified and encircled with a vessel loop. Type Ia endoleak at intraoperative DUS examination was present (Fig 4; Supplementary Video 1 (online only)). External proximal aortic neck banding was performed by creating a retroaortic tunnel immediately below the renal arteries. To limit the possible damage to the lumbar veins for the creation of the retroarctic tunnel, an iliac clamp was used, which reduced the maneuvers to encircle the neck and limited the manipulations of the retroaortic space; a 28-mm woven Dacron band was placed (Fig 5).

**Fig 6:**
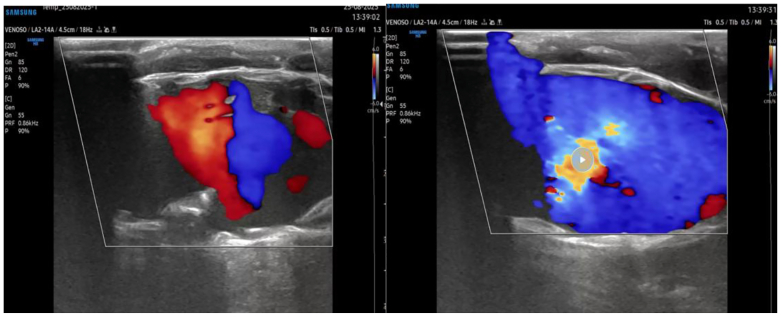
The disappearance of flow between the prosthesis and the aneurysm sac is appreciated; the endoleak is no longer present.

**Fig 7:**
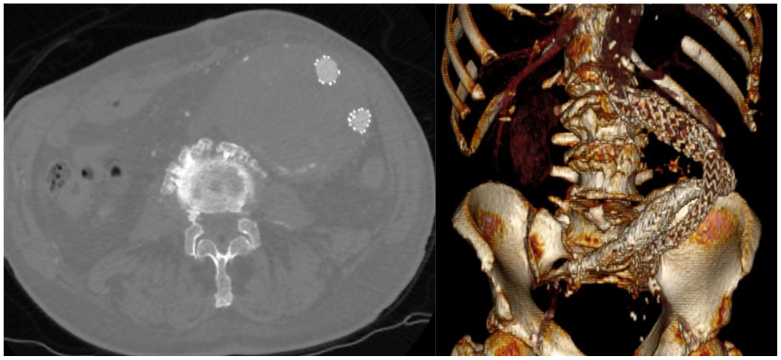
MIP and SSD reconstructions show the patency of the stent graft and no sign of any endoleak.

The anterior edges of the band were secured with 2-0 polypropylene sutures. The ideal diameter for sutures was considered to be the one reached when the first suture stitch was tightened, proximally. At that moment, the diameter was perceived to be reduced, as if an “accordion” had been completely closed. The suture was then completed by reaching the opposite end and moving back up with the same thread, creating a double overlock. The total operative time was 150 minutes. Final intraoperative DUS demonstrated complete resolution of the type IA endoleak and exclusion of the aneurysm sac (Fig 6; Supplementary Video 2 (online only)). The postoperative course and the follow-up at 8 months were uneventful. Follow-up imaging confirmed persistent exclusion of the aneurysm sac and no recurrence of endoleak sac or flow abnormality in the renal artery (Fig 7). The patient gave consent for the application of the images and the reported clinical case.

## Discussion

In recent years, an increasing number of patients have required secondary interventions after EVAR, including both endovascular reinterventions and open surgical conversion. The reported incidence of open conversion ranges from 2.2% to 2.5%, with perioperative mortality between 6.2% and 9.9%, markedly higher than that associated with primary open repair.^4^ Endoleaks represent the leading indication for conversion, accounting for approximately 80% of cases, most commonly, type I and type II endoleaks.^5^

Type IA endoleak is among the most clinically significant complications after EVAR, as it results in persistent aneurysm sac pressurization and a substantially increased risk of aneurysm enlargement and rupture. Although EVAR provides excellent short-term outcomes, mid- and long-term follow-up has demonstrated that progressive changes in proximal neck anatomy may lead to loss of proximal sealing, even after initially successful procedures.

In the present case, a type IA endoleak developed 10 years after EVAR in an elderly patient with a large abdominal aortic aneurysm (10 cm). Progressive proximal neck dilatation and angulation have been identified as major mechanisms of late proximal sealing failure after EVAR.^6^ In this setting, persistence of the endoleak associated with sac enlargement mandates active treatment, as conservative management is considered unsafe. In our opinion, the increase in diameter, compared with the previous check, and the persistence of flow within the sac, not as retrograde flow from a type II endoleak, but direct as from a type I endoleak, represent a high risk of rupture, even higher than that of an intact aneurysm of the same diameter.

Secondary endovascular repair is generally regarded as the first-line treatment for type IA endoleak. In our patient, an advanced endovascular strategy was employed, including proximal aortic cuff extension, chimney stenting of the left renal artery, and deployment of EndoAnchors to improve proximal fixation. Despite initial angiographic improvement, early follow-up imaging demonstrated persistent endoleak with continued sac enlargement, indicating failure of secondary endovascular therapy. This finding highlights that, despite technically complex interventions, some type IA endoleaks remain refractory because of unfavorable anatomic and biomechanical conditions.

Under these circumstances, open surgical conversion represents the definitive therapeutic option. Traditional conversion requires complete endograft explantation and graft replacement and is associated with substantial morbidity and mortality, particularly in elderly and frail patients. Increasing evidence, however, supports partial or complete endograft preservation as a means of reducing surgical invasiveness while maintaining adequate clinical efficacy.

Within this context, external proximal aortic neck banding or banding has emerged as a valuable salvage strategy. Karkos et al demonstrated that complete endograft preservation combined with proximal suturing and aortic neck banding is feasible and effective, even in post-EVAR aneurysm rupture, enabling a limited open conversion.^7^ Similarly, Okazaki et al reported favorable outcomes using external banding techniques for high-flow endoleaks, supporting its role in selected cases.^5^

In the present case, proximal neck banding was performed through a mini-laparotomy by creating a retroaortic tunnel immediately below the renal arteries and placing a woven Dacron band. By externally modifying neck geometry, this technique improves circumferential graft apposition and reduces proximal flow channels without increasing radial stress on the endograft. Intraoperative DUS confirmed immediate and complete resolution of the type IA endoleak with total exclusion of the aneurysm sac. The use of real-time duplex assessment avoided the need for early postoperative CTA, allowing follow-up imaging to be safely deferred.

This case supports proximal aortic neck banding as a valuable therapeutic option after failure of secondary endovascular repair, particularly in high-risk patients with complex proximal neck anatomy. Key advantages include endograft preservation, reduced operative time, avoidance of complex aortic clamping, lower procedural invasiveness, and reduction of contrast use compared with conventional open conversion. Although long-term durability data remain limited, proximal aortic neck banding appears to be an effective and reproducible salvage strategy in carefully selected patients.

## Conclusions

This case demonstrates that aortic neck banding with endograft preservation is a safe and effective option for the treatment of type IA endoleak refractory to endovascular therapy. Intraoperative DUS enabled real-time confirmation of endoleak resolution and absence of active aneurysm sac perfusion, obviating the need for immediate postoperative CTA. This approach was particularly advantageous in an elderly patient with chronic kidney disease. Further studies with longer follow-up are warranted to confirm the durability of this technique in larger patient cohorts.

## Funding

None.

## Disclosures

None.

## Supplementary data

Supplementary Video 1 In the central part of the image, color allows observation of the flow inside the endograft, downstream from the proximal neck. In the upper part, flow is present between the aneurysm wall and the endograft. Before bending, the endoleak is present.

Supplementary Video 2 The disappearance of flow between the wall and the endograft is visible. Flow in the endograft is preserved. The bending has been performed, and the endoleak has disappeared.
